# Evaluation of skin dose calculation factors in interventional fluoroscopy

**DOI:** 10.1002/acm2.12725

**Published:** 2019-09-30

**Authors:** Matthew C. DeLorenzo, Allen R. Goode

**Affiliations:** ^1^ Department of Radiology and Medical Imaging UVA Health Charlottesville VA USA

**Keywords:** dose, factors, fluoroscopy, radiation, RDSR, skin

## Abstract

**Purpose:**

The purpose of this study was to measure fluoroscopic dose calculation factors for modern fluoroscopy‐guided interventional (FGI) systems, and to fit to analytical functions for peak skin dose (PSD) calculation.

**Methods:**

Table transmission factor (TTF), backscatter factor (BSF), and a newly termed kerma correction factor (KCF) were measured for two interventional fluoroscopy systems. For each setup, air kerma rates were measured using a small ionization chamber in fluoroscopic service mode while selecting kVp, copper (Cu) filter thickness, incident angle, and x‐ray field size at the assumed patient skin locations. Angle dependency on KCF was measured on the GE system at isocenter for angles of 0, 15, 30, and 40 degrees, using a range of kVp, Cu filters, and one field size. An analytical equation was created to fit the data to facilitate PSD calculation.

**Results:**

For the GE system, oblique incidence measurements show KCF decreased by about 2%, 8%, and 13% for incident angles of 15, 30, and 40°, respectively, relative to KCF at 0 degree. The GE and Siemens systems' KCFs ranged from 0.89 to 1.45, and 0.64 to 1.44, respectively. The KCFs increased with a power of field size, and generally increased with kVp and Cu filter. The average percentage difference between TTF × BSF × *f* and KCF was 16% at normal incidence. The KCF data were successfully fitted to function of angle, field size, kVp, and Cu filter thickness using seven parameters, with an average R‐squared value of 0.98 and maximum percentage difference of 6.0%.

**Conclusions:**

This study evaluated scatter factors for two fluoroscopy systems, and dependencies on angle, kVp, Cu filter, and field size, with emphasis on under table beam orientations. Analytical fitting of the data with exposure parameters may facilitate PSD calculations, and more accurately determine the potential for radiation‐induced skin injury.

## 1 INTRODUCTION

Fluoroscopically guided intervention (FGI) uses x‐ray image guidance to perform minimally invasive procedures on patients affected by a range of medical conditions.^1^ In response to high patient skin radiation doses,^2^, ^3^, ^4^ fluoroscopy equipment manufacturers have implemented strategies for reducing radiation used during procedures.^5^, ^6^ Radiation use may now be monitored during and after the procedure and quantified using beam on time, reference point air kerma (*K_a,r_*), and kerma–area product (KAP). Some research groups have investigated real‐time and postprocedure radiation skin dose mapping.^7^, ^8^, ^9^ The National Council on Radiation Protection and Measurements (NCRP) suggests using the calculated peak skin dose (PSD) as a metric for managing prompt and latent skin reactions to radiation exposure.^10^

An equation to calculate patient skin dose is suggested by NCRP Report 168^10^ and Jones AK^11^ is given by:

(1) $$D_{\text{skin}}=K_{a,r}{\left(\frac{d_{r}}{\text{SSD}}\right)}^{2}\text{TTF}\times \text{BSF}\times f$$

where *K_a,r_* is the air kerma reported at the interventional reference point,^6^ *d_r_* is the source to reference point distance, SSD is the source‐to‐skin distance, TTF is the table transmission factor, BSF is backscatter factor of soft tissue,^12^ and *f* is the dose conversion factor from air to soft tissue.^13^ This approach has been used by research groups^7^, ^8^, ^9^ studying skin dose mapping and has been shown to be accurate with using the fluoroscope’s reported reference air kerma, and measurements of BSF and TTF.

Backscatter factors have been calculated using Monte Carlo simulations for a variety of x‐ray beam qualities,^14^ and for filtered x‐ray beams intended to reduce patient dose.^15^ More recently, Wunderle et al measured BSFs for a modern fluoroscope that employs copper (Cu) filtration, using polymethyl methacrylate (PMMA) blocks and an over‐table x‐ray tube setup.^16^ The authors found that BSF depended on kVp, added Cu filter thickness, and x‐ray field size, and ranged from 1.18 (using 60 kVp, 0.0 mm Cu, 11 cm field of view) to 1.58 (using 80 kVp, 0.9 mm Cu, 42 cm field of view). Polymethyl methacrylate is a common phantom material and surrogate for soft tissue,^17^ but it has been demonstrated that the scatter properties are not identical,^15^, ^18^ and a correction is necessary to account for these differences. Corrected BSF measurements may be readily applied to laterally oriented x‐ray beams, but may not necessarily apply to under‐table exposures due to the attenuation and forward scatter from the table and pad.

The patient support has been shown to affect image quality for automatic brightness control fluoroscopy units.^19^ The energy spectrum incident on the patient skin for under‐table exposures would therefore differ from those oriented in the lateral direction. DeLorenzo et al. showed that the patient support attenuates the x‐ray beam and becomes a source of forward scatter, and some combinations of table and pad can reduce the transmission of air kerma to 60% of its original value.^20^ With the presence of the patient support in mind, we hypothesize that PSD calculations would benefit from measuring BSF and TTF simultaneously to produce a single value for x‐ray beam orientations that intercept the patient support. We call this value the kerma correction factor (KCF), which intends to convert the inverse square corrected *K_a,r_* to *D*_skin_ by taking into account the forward scatter and potential spectral changes due to the patient support.

(2) $$D_{\text{skin}}=K_{a,r}{\left(\frac{d_{r}}{\text{SSD}}\right)}^{2}\text{KCF}$$

Two interventional fluoroscopic x‐ray systems were used in this study: the General Electric (GE) Innova 2100 with Omega V table, and the Siemens Artis Zee with Siemens Tabletop Narrow (CARD) table. Nominal values for attenuation and measured dimensions for these tables are provided in previous work.^20^ The support pad is by Burlington Medical (model MA‐09‐3979, Newport News, VA, USA) and is made of 100% high resilient polyurethane foam.

The goal of this study is to present a comprehensive dataset using two fluoroscopic systems to accurately convert reported air kerma to skin dose. Air kerma measurements at the surface of PMMA phantoms are taken as functions of kVp, added Cu filter thickness, x‐ray field size, and incident angle. The dataset in this work will enable skin dose calculation from x‐ray beams intercepting the patient support table directly KCF, at oblique angles using an angle correction factor *F*_θ_, and for lateral x‐ray beam orientations BSF from information available in digital imaging and communications in medicine radiation dose structured reports (RDSRs). Factors are fitted to analytical equations for more automated implementations of patient skin dose calculation.

## 2 MATERIALS AND METHODS

### 2.1 KCF measured at zero degrees

**Figure 1:**
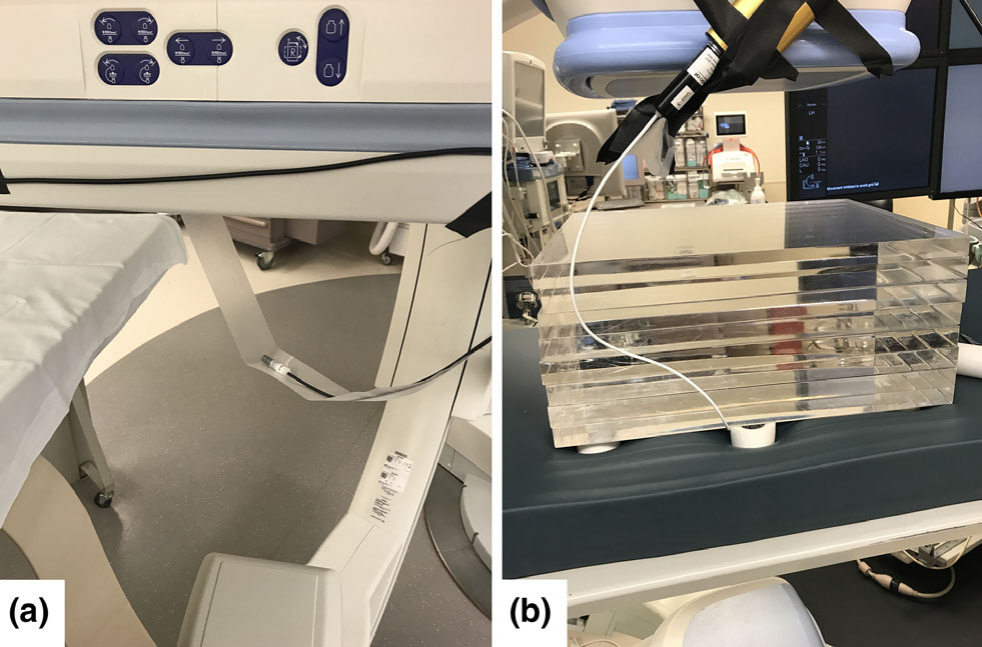
(a) Setup 1: Air kerma rate measured without patient support using the RadCal 10x6‐0.6 ion chamber. (b) Setup 2: Under‐table setup with influence of backscatter and table transmission to measure KCF for 20 cm (pictured) blocks of PMMA. KCF, kerma correction factor; polymethyl methacrylate.

Using a 0.6 cm^3^ volume ion chamber (RadCal 10x6‐0.6), the KCF was measured as a function of kVp, Cu filter thickness, and x‐ray field size. The GE Innova 2100 fluoroscope was operated in service mode using the largest available focal spot size (1.2 mm), 45 mA, 10 ms pulse width, and 30 pulses per second, for 60–120 kVp, in 10 kVp increments, for six added filter selections (0.0, 0.1, 0.2, 0.3, 0.6, and 0.9 mm Cu) and three field sizes (7 × 7 cm^2^, 10 × 10 cm^2^, and 13.5 × 13.5 cm^2^) measured at the ion chamber location with lead rulers. The Siemens Artis Zee fluoroscope was operated using fewer kVp increments (60, 90, 120 kVp) and Cu filters (0.0, 0.3, 0.9 mm Cu), and four field sizes (5 × 5 cm^2^, 10 × 10 cm^2^, 15 × 15 cm^2^, and 21.6 × 21.7 cm^2^). Air kerma rates were recorded with the chamber suspended freely in air [Fig. 1(a), setup 1], and at the assumed patient skin location between the patient support and a 20‐cm PMMA phantom [Fig. 1(b), setup 2]. The chamber was centered in the x‐ray beam, in the “torso” region of the patient support. The KCF was computed by multiplying the air to tissue dose conversion factor (f‐factor) by the ratio of exposure rates from setup 2 by inverse square law corrected exposure rates from setup 1 (see Fig. 1),

(3) $$\text{KCF}=f\times B_{\text{PMMA}}^{\text{st}}\times \frac{K_{\text{setup}\;2}}{K_{\text{setup}1}}\times {\left(\frac{{\text{SCD}}_{1}}{{\text{SCD}}_{2}}\right)}^{2}$$

where *f* is the f‐factor*,* $B_{\text{PMMA}}^{\text{st}}$ is the ratio of backscatter factors of soft tissue to PMMA, *K*_setup 1_ and *K*_setup 2_ are kerma rates measured for a given exposure parameter configuration, and SCD_1_ (83 cm) and SCD_2_ (81.5 cm) are source‐to‐chamber distances for setup 1 and setup 2, respectively. The f‐factor is tabulated with half value layer (HVL) by the International Council on Radiation Protection (ICRP),^12^ and was fitted to a linear function of HVL (*R* ^2^ = 0.94). The HVL for each configuration of kVp and Cu filter was measured separately for the Siemens and GE fluoroscopes using a RadCal Accu‐gold AGMS‐D + solid‐state multisensory, and the f‐factor for each beam quality setting was calculated and used in Eq. 3. To account for differences in scatter properties between PMMA and soft tissue, backscatter factors from Ref. 15 were compared. For a variety of kVp, filters, field sizes, and HVLs investigated, the average ratio of backscatter factors of ICRU soft tissue and PMMA was 0.943 and standard deviation 0.006. A constant value of 0.943 is therefore applied to all measurements with PMMA as the backscatter phantom material. For all air kerma rate measurements in this work, a lead blocker was inserted into the image receptor grid holder to minimize scatter from the flat panel detector and to protect the detector from excessive radiation intensity.

### 2.2 Dependency of KCF on incident angle

The GE Innova 2100 system was used to measure the dependence of incident angle on KCF. The system’s radiation isocenter was found using a fiducial marker and multiple views, and was measured to be 70 cm from the x‐ray source. The RadCal 10x6‐60 ion chamber was placed on the patient support pad at the system’s isocenter and secured with tape. Next, slabs totaling 20‐cm PMMA were placed tightly above the chamber and support pad at the assumed patient skin location. Air kerma rates were measured using all configurations of kVp and Cu filter in Section II.A.1. The lateral gantry angles of 0° (corresponding to the PA view), 15°, 30°, and 40° were used with a nominal field size of 11.8 × 11.8 cm^2^ at the chamber location. An angle factor, *F_θ_* was calculated as,

(4) $$F_{\theta }=\frac{K_{\theta }}{K_{0^{\circ }}}$$

to correct the KCF measurements at 0° to account for obliquely incident beams.

### 2.3 Backscatter factor

Backscatter factor was measured using both fluoroscopes to estimate skin doses for laterally oriented beams. The patient support pad was removed and the fluoroscope was oriented in the lateral direction at 90°, without the x‐ray field intercepting the patient support. A ceiling‐mounted protective face shield was positioned above the x‐ray field and was used as a platform to suspend the RadCal 10x6‐0.6 ion chamber. The ion chamber was carefully centered in the x‐ray field, 90 cm from the x‐ray focal spot, to achieve a rotated version of setup 1. Air kerma rates were measured for all previous exposure parameter configurations of kVp, added Cu filtration, and field size, using a 20‐cm thick PMMA phantom positioned upright directly behind the suspended ion chamber. Backscatter factor for each exposure parameter configuration without the influence of the patient support was found by dividing exposure rates with and without PMMA blocks behind the chamber, and correcting for scatter property differences between ICRU soft tissue and PMMA.

(5) $$\text{BSF}=\frac{K_{\text{acrylic}}}{K_{\text{air}}}\times B_{\text{PMMA}}^{\text{st}}$$

### 2.4 Table transmission factor

Table transmission factor was measured to compare the quantity TTF × BSF × *f* to KCF. Using the suspended ion chamber setup in Fig. 1, air kerma rates were measured with and without the patient support in the beam path. For the GE fluoroscope, the 13.5 × 13.5 cm^2^ field size was used with the 10x6‐60 ion chamber, for all kVp and Cu filter combinations. Using the 10x6‐0.6 ion chamber, table transmission factor was calculated for each kVp (60, 90, 120 kVp), Cu filter (0.0, 0.3, 0.9 mm Cu), and field size (5 × 5 cm^2^, 10 × 10 cm^2^, 15 × 15 cm^2^, and 21.6 × 21.7 cm^2^) combination by dividing the respective air kerma rates measured with and without the table and pad in the beam path.

### 2.5 Analytical data fitting

The measured KCF and BSF were fitted to an analytical function of kVp, Cu filter, field size, and incident angle. The effect of incident angle was employed as a multiplicative factor to correct data measured for x‐ray beams at normal incidence. The final expression will take the form,

(6) $$\text{KCF},\text{BSF}=B_{1}\left(k,c,\theta \right)B_{2}\left(k,c,x\right)$$

where *B*_1_ represents the dependency on incident angle, and *B*_2_ describes the dependency on kVp, Cu filter, and x‐ray field size. Matlab (Mathworks Inc., Natick, MA, USA) least squares nonlinear curve fitting was used to fit the measured KCF and BSF data to a model, and a representative equation with the highest coefficient of determination (*R* ^2^) and lowest maximum percentage difference between predicted and measured values was reported.

### 2.6 Phantom simulation

**Figure 2:**
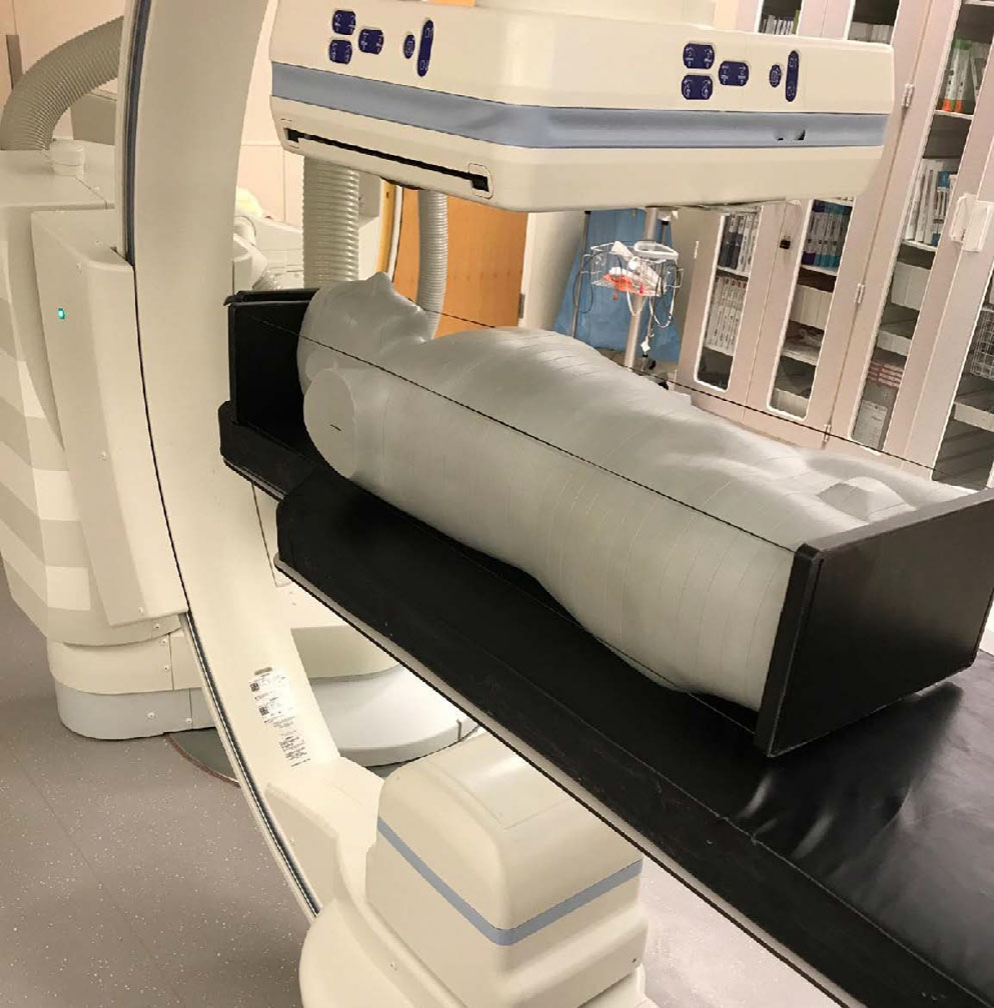
Anthropomorphic phantom experiment setup using 12 radiation events with different kVp, Cu filter, field size, and angle. Dose to air was measured under the torso of the phantom with a Radcal 10x6‐0.6 ion chamber, and corrected using $B_{\text{PMMA}}^{\text{st}}.$ The measured surface dose was compared to results predicted by Eqs (7), (8), (9) and Eq 1. Results are shown in Table 6. PMMA, polymethyl methacrylate.

An interventional fluoroscopic procedure was simulated using an anthropomorphic (adult male), tissue equivalent^21^ body phantom (ATOM phantom, CIRS, Norfolk, VA, USA) to assess the accuracy of Eq. 6, and to compare the approach of using KCF vs TTF × BSF × *f*. Using the Siemens Artis Zee system, the phantom was positioned on the patient support supine, and the 10x 6‐0.6 Radcal ion chamber was placed beneath the phantom’s thoracic spine region, seen in Fig. 2. Measured ion chamber doses to air were corrected using f‐factors for soft tissue based on measured HVL data. The phantom was scheduled in the worklist of the fluoroscope to allow retrieval of the case’s RDSR from Radimetrics (Bayer Healthcare, Whippany, NJ, USA). The lead rulers were taped to the image receptor to measure field size, and a tape measure and magnification factors were used to determine the field size at the phantom skin location for each x‐ray beam.

**Figure 3:**
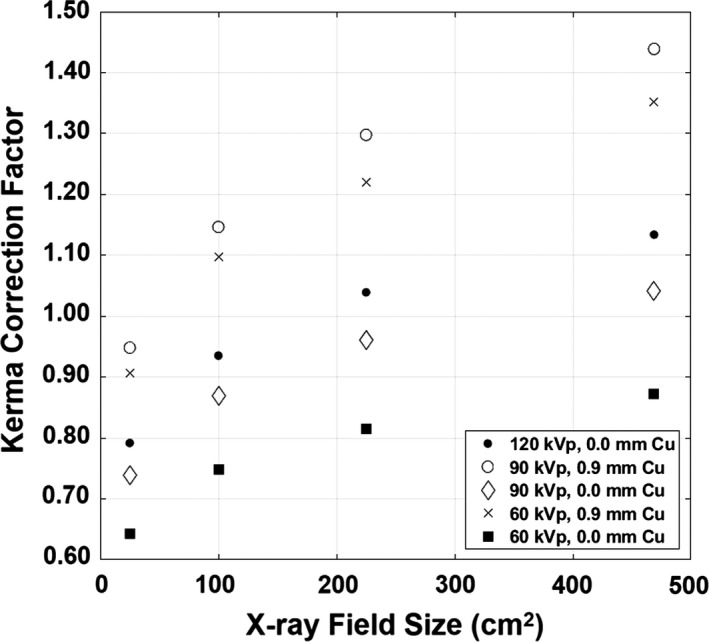
KCF as a function of x‐ray field size for the Siemens Artis Zee system. KCF increased with field size due to the increased scatter from the edges of the PMMA phantom. The field size dependence was more pronounced as the added Cu filter thickness increased. Field sizes were measured at the ion chamber location. KCF, kerma correction factor; PMMA, polymethyl methacrylate.

A total of 12 radiation events were used in the simulation, each with a different combination of kVp, Cu filter, x‐ray field size, incident angle, and reference point air kerma. Source to image distance (SID) was 120 cm for all events, with lead rulers 117 cm from the source. The operator selected dose program, table height, and magnification mode, allowing the fluoroscope to determine kVp, mA, pulse width, and Cu filter. For each radiation event (kVp, Cu filter, and field size), BSF and TTF were measured separately, and HVLs for each beam were recorded using the AGMS‐D+ solid state detector to determine f‐factors. The dose to the skin for each beam was estimated using KCF (eq 2) and calculated from measured BSF, TTF, and f‐factor estimated using HVL (eq 1), and compared to the ion chamber measurements Fig. 3.

## 3 RESULTS

### 3.1 KCF at zero degrees

**Figure 4:**
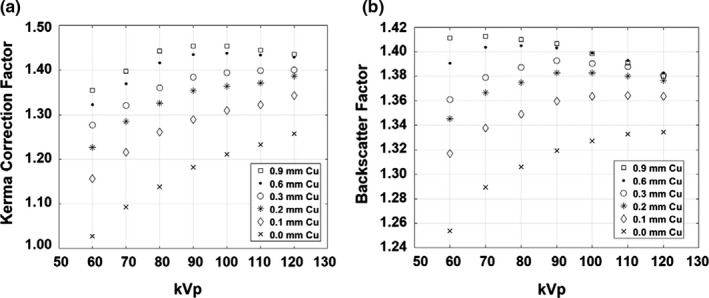
KCF (a) and BSF (b) with 20‐cm thick PMMA phantom and GE Innova 2100, using RadCal 10x6‐0.6 ion chamber and 13.5 × 13.5 cm2 field size. KCF generally increases with kVp and added Cu filter for all but the highest energy x‐ray beams. BSF has a different relationship with kVp and added Cu filter, reaching a maximum value of 1.41 at 60 kVp and 0.9 mm Cu. BSF, backscatter factor; KCF, kerma correction factor; PMMA, polymethyl methacrylate.

Figure 4 shows KCF and BSF from the GE system as a function of kVp and Cu filter for the 20‐cm thick PMMA phantom. KCF values for the GE system and Siemens system are shown in Tables 1 and 2, respectively. KCF tended to increase with kVp and Cu filter, and increased as a power of field size. KCF for both fluoroscopes was smallest at 60 kVp and 0.0 mm Cu filter, and largest at 90 kVp and 0.9 mm Cu. KCF decreased after 90 kVp for 0.6 and 0.9 mm Cu despite the f‐factor increasing monotonically.

**Table 1:** GE Innova 2100 KCF and BSF for three field sizes at ion chamber location, using 20‐cm PMMA phantom.

Field Size (cm2)	kVp/mm Cu	KCF						BSF					
		0	0.1	0.2	0.3	0.6	0.9	0	0.1	0.2	0.3	0.6	0.9
7 × 7	60	0.89	0.98	1.02	1.04	1.07	1.08	1.17	1.20	1.22	1.23	1.25	1.27
	70	0.93	1.01	1.06	1.08	1.10	1.10	1.18	1.21	1.22	1.23	1.25	1.25
	80	0.96	1.04	1.08	1.10	1.15	1.15	1.19	1.21	1.22	1.23	1.24	1.24
	90	1.00	1.06	1.10	1.12	1.15	1.16	1.20	1.22	1.22	1.22	1.23	1.22
	100	1.02	1.08	1.11	1.13	1.15	1.16	1.20	1.21	1.22	1.22	1.22	1.22
	110	1.03	1.09	1.12	1.14	1.16	1.16	1.20	1.21	1.21	1.21	1.21	1.20
	120	1.05	1.11	1.13	1.14	1.16	1.16	1.20	1.20	1.20	1.20	1.20	1.19
10 × 10	60	0.96	1.07	1.12	1.15	1.18	1.20	1.22	1.26	1.31	1.33	1.37	1.39
	70	1.01	1.11	1.16	1.19	1.22	1.23	1.26	1.29	1.32	1.33	1.36	1.37
	80	1.05	1.14	1.19	1.22	1.26	1.28	1.27	1.30	1.32	1.33	1.36	1.36
	90	1.09	1.17	1.22	1.24	1.28	1.29	1.28	1.31	1.33	1.34	1.35	1.34
	100	1.11	1.19	1.23	1.25	1.28	1.29	1.29	1.31	1.32	1.33	1.34	1.33
	110	1.13	1.20	1.24	1.25	1.28	1.29	1.29	1.30	1.31	1.32	1.32	1.31
	120	1.15	1.22	1.25	1.26	1.28	1.28	1.29	1.30	1.31	1.31	1.31	1.29
13.5 × 13.5	60	1.03	1.16	1.23	1.28	1.32	1.35	1.29	1.34	1.37	1.41	1.45	1.47
	70	1.09	1.22	1.29	1.32	1.37	1.40	1.31	1.36	1.40	1.42	1.44	1.45
	80	1.14	1.26	1.33	1.36	1.42	1.44	1.33	1.38	1.41	1.41	1.43	1.43
	90	1.18	1.29	1.35	1.38	1.44	1.45	1.35	1.39	1.41	1.41	1.42	1.41
	100	1.21	1.31	1.36	1.39	1.44	1.45	1.35	1.39	1.41	1.41	1.41	1.40
	110	1.23	1.32	1.37	1.40	1.43	1.45	1.36	1.38	1.40	1.39	1.40	1.38
	120	1.26	1.34	1.39	1.40	1.43	1.44	1.36	1.38	1.39	1.39	1.39	1.37

BSF, backscatter factor; KCF, kerma correction factor; PMMA, polymethyl methacrylate.

**Table 2:** Siemens Artis Zee KCF and BSF, using 5 × 5, 10 × 10, 15 × 15, and 21.6 × 21.7 cm^2^ field sizes and 20‐cm PMMA phantom.

Field Size (cm^2^)	kVp/mm Cu	KCF			BSF		
		0	0.3	0.9	0	0.3	0.9
5 × 5	60	0.64	0.84	0.91	1.01	1.06	1.06
	90	0.74	0.90	0.95	1.03	1.05	1.06
	120	0.79	0.92	0.95	1.03	1.04	1.04
10 × 10	60	0.75	1.00	1.10	1.11	1.20	1.22
	90	0.87	1.09	1.15	1.14	1.22	1.22
	120	0.94	1.11	1.14	1.15	1.20	1.19
15 × 15	60	0.82	1.11	1.22	1.15	1.28	1.32
	90	0.96	1.22	1.30	1.21	1.31	1.33
	120	1.04	1.25	1.28	1.22	1.30	1.29
21.6 × 21.7	60	0.87	1.22	1.35	1.17	1.32	1.36
	90	1.04	1.35	1.44	1.24	1.38	1.39
	120	1.13	1.37	1.42	1.27	1.34	1.34

BSF, backscatter factor; KCF, kerma correction factor; PMMA, polymethyl methacrylate.

KCF most strongly depended on field size and the results show that field size should be taken into account when calculating patient skin dose. KCF for the Siemens system at 90 kVp and 0.9 mm Cu filter can range from 1.06 for the smallest field size measured (25 cm^2^) to 1.39 for the largest field size available on the system at the assumed patient skin location (469 cm^2^). KCF tended to increase faster as the HVL of the kVp/filter combination increased.

The GE system’s HVLs for all kVp/Cu filter combination were higher than the Siemens system, seen in Table 3, particularly for low values of Cu filter thickness. The trend for both fluoroscopes is that KCF increases with HVL. This would result more penetration through the table and pad, and a higher scatter fraction off the PMMA toward the detector. Kerma correction factor for the GE system was consistently larger than for the Siemens system.

**Table 3:** Table transmission factor for the Siemens table and Burlington pad.

Field Size (cm^2^)	kVp/mm Cu	TTF		
		0	0.3	0.9
5 × 5	60	0.52	0.65	0.70
	90	0.59	0.70	0.73
	120	0.63	0.72	0.74
10 × 10	60	0.54	0.68	0.72
	90	0.61	0.73	0.76
	120	0.65	0.75	0.77
15 × 15	60	0.56	0.71	0.74
	90	0.63	0.75	0.78
	120	0.68	0.77	0.80
21.6 × 21.7	60	0.59	0.73	0.78
	90	0.66	0.78	0.81
	120	0.70	0.80	0.83

TTF, Table transmission factor.

### 3.2 KCF dependency on incident angle

**Figure 5:**
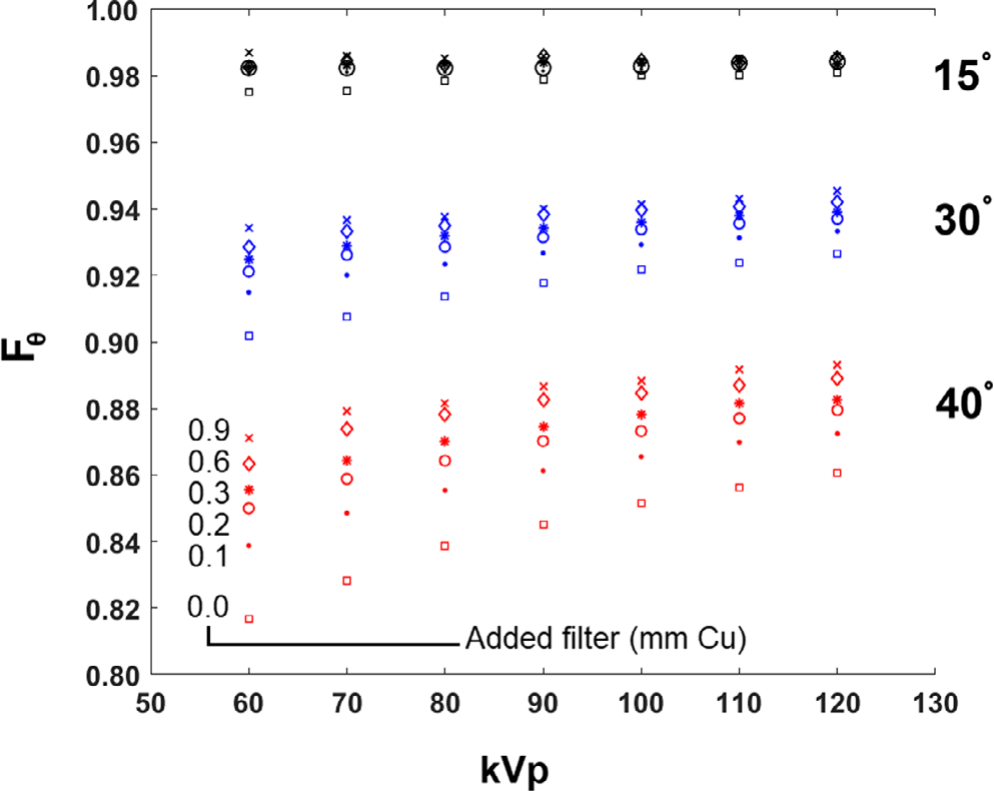
Oblique correction for KCF. *Fθ* plotted for three incident angles (15°, 30°, and 40°) with respect to 0°, found using the GE system with Omega V table and Burlington pad, and a medium field size (13.5 × 13.5 cm2). KCF decreases by about 2%, 7%, and 14% for incident angles of 15°, 30°, and 40°, respectively. GE, General Electric; KCF, kerma correction factor.

The relative fraction of transmission, *F*_θ_, between 0° incidence and 15°, 30°, and 40° incidence ranged from 0.975 to 0.987, 0.902 to 0.946, and 0.817 to 0.893, respectively. Figure 5 shows a plot of *F*_θ_ as a function of kVp for 15°, 30°, and 40°, for six added Cu filters. *F*_θ_ increases with kVp, added Cu filter, and decreases with incident angle. *F*_θ_ is used as a multiplicative factor to correct KCF for obliquely incident gantry angles, θ, where 0° indicates normal incidence of the x‐ray beam’s central ray into the patient.

### 3.3 Backscatter factor

The BSF of the GE system and 13.5 × 13.5 cm^2^ field size is shown in [Fig. 4(b)]. Full BSF values for the GE system and Siemens system are shown in Tables 1 and 2, respectively. BSF generally increased with Cu filter thickness. For smaller values of Cu filter thickness, BSF increased with kVp, and then decreased for larger values of filter thickness. BSF for the GE system was largest at 60 kVp and 0.9 mm Cu, and BSF for the Siemens system was largest at 90 kVp and 0.9 mm Cu filter. This difference in shape and maximum value highlights the differences in energy spectrum properties between the two systems.

### 3.4 Table transmission factor

Table transmission factors for the GE and Siemens systems can be found in Tables 3 and 4. The relatively thick Burlington pad was 8.0 cm uncompressed and resulted in significant attenuation of the primary beam. For the Siemens system, with its relatively softer beam quality, the patient support transmitted 52–83% of the primary radiation. The GE system transmitted 62–82% of the primary radiation.

**Table 4:** Table transmission factor for the GE table and Burlington pad, using a 13.5 × 13.5 cm^2^ field size at the ion chamber location.

kVp/mm Cu	TTF					
	0.0	0.1	0.2	0.3	0.6	0.9
60	0.62	0.68	0.71	0.73	0.75	0.77
70	0.64	0.70	0.73	0.75	0.77	0.78
80	0.67	0.72	0.75	0.76	0.79	0.80
90	0.68	0.74	0.76	0.77	0.80	0.80
100	0.70	0.75	0.77	0.78	0.80	0.81
110	0.71	0.76	0.78	0.79	0.81	0.81
120	0.72	0.76	0.78	0.79	0.81	0.82

TTF, Table transmission factor.

### 3.5 Modeling of the broad beam transmission

Several equation forms were investigated to accurately fit the measured data, and our selection criteria focused on maximizing the total *R* ^2^ while minimizing the largest percentage difference between measured and fitted values. The transmission data were successfully fitted to the following equations,

(7) $$F_{\theta }\left(k,c,\theta \right)=q_{1}\left(c-q_{2}\right)\theta^{2}+q_{3}\theta +q_{4}kc+1$$

(8) $$\text{KCF}\left(x,k,c\right)=F_{\theta }\times \left[p_{1}k+p_{2}{\left(c+1\right)}^{p_{3}}\right]\times x^{\left(p_{4}k+p_{5}{\left(c+1\right)}^{p_{6}}\right)}+p_{7}c$$

(9) $$\text{BSF}\left(x,k,c\right)=F_{\theta }\times \left[p_{1}k+p_{2}{\left(c\right)}^{p_{3}}\right]\times x^{\left(p_{4}k+p_{5}{\left(c+1\right)}^{p_{6}}\right)}+p_{7}$$

where *θ* was the incident angle of the central ray (degrees), *x* was the x‐ray field size at the point of measurement (m^2^), *k* was the kVp, and *c* was the added Cu filter thickness (mm). The coefficients for each patient support, percentage differences from measured values, and *R* ^2^ values are shown in Table 5.

**Table 5:** Fitting parameters to be used with Eqs. (7), (8), (9) to estimate KCF and BSF for GE, Siemens, and fluoroscope used in Ref. 16. KCF and BSF values were corrected for ICRU soft tissue, except Ref. 16.

Term	GE		Siemens		Ref. 16
	KCF	BSF	KCF	BSF	BSF (PMMA)
*p*_1_	6.65E‐03	1.65E‐02	9.99E‐03	9.51E‐03	1.24E‐02
*p*_2_	1.29	1.22	5.27E‐01	5.45E‐01	7.90E‐01
*p*_3_	1.47	7.84E‐01	1.80	4.74E‐01	5.79E‐01
*p*_4_	1.45E‐04	2.10E‐03	7.96E‐04	2.01E‐03	1.77E‐03
*p*_5_	1.08E‐01	1.28E‐01	3.63E‐02	6.06E‐02	7.62E‐02
*p*_6_	2.25	8.35E‐01	3.66	9.92E‐01	9.50E‐01
*p*_7_	9.08E‐01	9.28E‐01	8.74E‐01	8.44E‐01	9.62E‐01
*R* ^2^	0.99	0.99	0.99	0.97	0.98
Mean difference (%)	1.34	0.72	1.77	1.84	1.46
Max difference (%)	4.77	2.32	5.99	4.99	3.05
Oblique Term KCF					
*q*_1_	2.508E‐05				
*q*_2_	3.934				
*q*_3_	−6.142E‐04				
*q*_4_	1.025E‐05				

BSF, backscatter factor; KCF, kerma correction factor; PMMA, polymethyl methacrylate.

### 3.6 Phantom surface dose

The total accumulated dose and dose per radiation event measured with the ion chamber were compared to the values predicted by eq 8 (KCF fitting method), and using eq 1 (measured TTF × BSF × *f* method). A summary of the phantom results is shown in Table 6. The total dose to soft tissue recorded by the ion chamber for 12 beams was 909 mGy, while the dose predicted by the KCF method and fitting eq 8 was 836 mGy, and the total dose calculated using separately measured BSF and TTF with eq 1 was 720 mGy. Dose calculated using separately measured TTF × BSF × *f* was consistently lower than the measured dose for all 12 beams.

**Table 6:** Twelve beams from the Siemens fluoroscope were used to simulate an interventional procedure on an anthropomorphic phantom torso, and predicted skin dose from eqn 8 and from separately measured BSF and TTF. F‐factors were calculated from measured HVL, and ion chamber doses to air were corrected using the same f‐factors. Dose program, table height, and field size were varied by the operator, while kVp, mA, pulse width, and Cu filter were determined by the fluoroscope.

Beam	kVp	Cu (mm)	FS (cm^2^)	Angle	DAP (mGy cm^2^)	SSD (cm)	KCF	BSF × TTF × *f*	D_meas_ (mGy)	D (Eqs. 8, mGy)	D (Eqs. 1, mGy)
1	68	0.3	90	0°	7988	75	1.02	0.90	90.2	90.3	80.1
2	78	0.2	90	0°	7064	75	0.98	0.90	80.1	77.1	70.5
3	96	0	90	0°	21455	75	0.87	0.75	220.9	208.2	179.0
4	69	0.3	315	0°	14807	75	1.18	1.01	58.1	55.2	47.4
5	78	0.9	315	0°	2586	75	1.32	1.15	11.2	10.8	9.5
6	79	0.1	315	0°	24955	75	1.04	0.92	93	82.2	73.1
7	81	0.6	97	35°	1775	78	1.11	0.99	20.2	17.8	15.8
8	71	0.2	97	35°	5912	78	0.97	0.87	59.7	51.1	45.9
9	89	0	97	35°	12068	78	0.86	0.73	110	91.7	77.7
10	68	0.3	434	0°	17662	88	1.22	1.01	52.1	49.6	41.0
11	68	0.3	434	0°	534	88	1.22	1.01	1.6	1.5	1.2
12	81	0	434	0°	44584	88	0.97	0.76	111.4	100.0	78.4
								Sum:	908.5	835.7	719.5

BSF, backscatter factor; HVL, half value layer; SSD, source‐to‐skin distance; TTF, Table transmission factor.

## 4 DISCUSSION

For both the GE and Siemens systems, the table and pad had a substantial effect on the measured air kerma rate. The tables and relatively thick support pad attenuated about 25% of the beam for moderate values of kVp and added Cu filter (Table 4). For unfiltered beams using low kVp, the patient support transmitted as little as 52% of the primary beam. For the GE system, using the 13.5 × 13.5 cm^2^ field size and 20‐cm PMMA phantom, the average percentage difference between TTF × BSF × *f* and KCF was 15.8% at normal incidence. For the Siemens system, average percentage differences were 13%–16% across the ranges of kVp, Cu filter, and field size. The setup used for measuring KCF more closely matches the clinical scenario for under‐table x‐ray source geometry, and the authors suggest KCF be used in lieu of TTF × BSF × *f* for x‐ray beams intercepting the patient support.

Wunderle et al. measured the BSF using a PMMA and an over‐table x‐ray source geometry. A slab of PMMA was machined to include a cavity to embed the chamber such that the point of measurement was at the assumed patient skin location, while the ion chamber in this work rested on the flat PMMA surface. Our measurement values were close those found by Wunderle et al, suggesting that the exact geometry of the scattering medium in relation to the chamber is not critical. Both experiments showed an increase in BSF with a power law dependency on field size, and Wunderle’s data were fitted reasonably well to eq 9, with *R* ^2^ = 0.98, a mean percentage difference of 1.46%, and a maximum percentage difference of 3.05% between fitted and measured values.

The angle factor, *F_θ_*, for the KCF showed the effect of incident angle on the kerma experienced at the assumed patient skin location, as a fraction of what was measured at normal incidence. *F_θ_* is consistent with the increased path length through the patient support with incident angle, and thus, a simple path length correction is a good approximation. This finding was also reported by Rana et al. 7 For angles of 30° and 40°, the entire x‐ray beam went through the patient support, but part of the beam impacted the side surface of the 20‐cm thick PMMA phantom. This decrease in *F_θ_* for larger angles resulting from sideways incidence onto the phantom would presumably become more pronounced with SSD for the same phantom, and is unclear how *F_θ_* would change with a rounded phantom that more closely approximates the body habitus of the patient.

The cumulative skin dose measurement from the anthropomorphic phantom experiment matched well with that predicted by the KCF fit equation (8% difference), and showed an improvement over separately measured correction factors (21%). Although the KCF formalism yielded an improvement, there are many uncertainties not accounted for in the phantom experiment. A major consideration was the curved phantom surface above the chamber which partially shrouded the point of measurement with scatter material. Johnson and Borrego et al noted that the entrance surface of anthropomorphic phantoms is typically flat as they lay on the support pad, regardless of body habitus.^9^ The ATOM phantom used in this simulation was rigid and had a recession in the lower spine area, which presumably affects the scatter geometry. The measured doses at this point were higher than the predicted values. Another consideration is the presence of bone directly behind the ion chamber, and lung in the periphery of the field of view. Heterogeneity of the human phantom is potentially a major source of uncertainty, but the use of KCF did yield a substantial improvement in the dose estimate compared to BSF × TTF × *f*.

**Figure 6:**
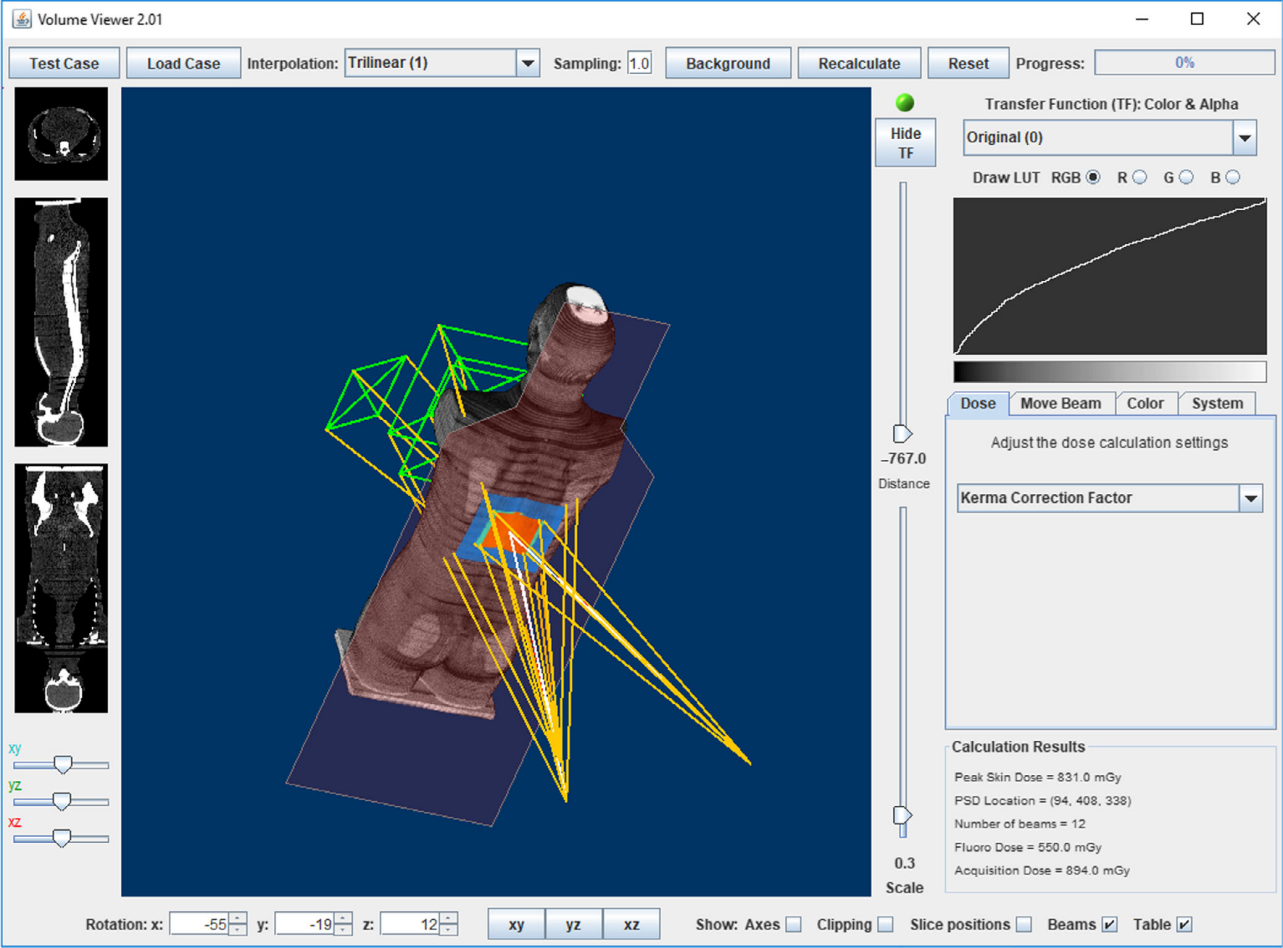
Twelve beam anthropomorphic phantom experiment simulated in custom peak skin dose calculation software. RDSR was converted by Radimetrics into spreadsheet format and imported into modified Volume Viewer. The resulting PSD calculation was performed using KCF for each radiation event. KCF, kerma correction factor; PSD, peak skin dose; RDSR, radiation dose structured reports.

In practice, one might use eqs (7), (8), (9) together with the radiation event information available in the RDSR. For example, KCF and BSF can be estimated easily for each radiation event from the RDSR, which contains kVp and Cu filter data, and effective angle of the beams’ central rays. The HVL can be estimated using Table 7, but does not affect f‐factor much. Dose to air at the patient skin location is estimated using inverse square corrections, reference point air kerma, and knowledge of table location, all of which are readily available. Field size at the skin can be calculated by multiplying the KAP by a magnification factor and dividing by reference point air kerma. Initially started at Massachusetts General Hospital (Boston, MA, USA), we use custom software at our institution derived from ImageJ and Volume Viewer plugin,^22^ shown in Fig. 6. The viewer was modified to volume render a CT image of the ATOM phantom, parse a spreadsheet of radiation event information, create virtual beams superimposed on the phantom, and sum the skin doses at each voxel calculated using eqs (7), (8), (9), or alternatively Eq 1. Other solutions are available in commercial software.

**Table 7:** Measured half value layers (mm Al eq) using the Radcal Accu‐gold AGMD‐D+ for selections of kVp and Cu filter thickness. All measurements were obtained under fluoroscopic service mode with the chamber unobstructed by the patient support.

kVp	Siemens X‐ray Source		
	Cu filter (mm)		
	0	0.3	0.9
60	2.37	5.01	6.75
90	3.47	7.45	9.79
120	4.63	9.19	11.48

kVp	GE X‐ray Source					
	Cu filter (mm)					
	0	0.1	0.2	0.3	0.6	0.9
60	2.80	3.86	4.68	5.19	6.14	6.86
70	3.30	4.56	5.53	6.17	7.26	7.99
80	3.76	5.20	6.30	6.96	8.23	9.05
90	4.24	5.81	6.99	7.70	9.02	9.87
100	4.73	6.39	7.64	8.36	9.71	10.67
110	5.20	6.95	8.24	8.95	10.31	11.22
120	5.65	7.50	8.78	9.47	10.84	11.74

The fit equation enables a more automated and beam‐specific consideration of PSD correction factors when used alongside the radiation event data sent from the fluoroscope. For every beam in the anthropomorphic phantom experiment, the ion chamber remained in the center of the field of view, consistent with the setups with PMMA used to measure KCF. This study assumes a uniform intensity across the x‐ray field, and one may expect a decrease in primary beam intensity and scatter for off‐axis ion chamber measurements.

A limitation of this study is the use of a rectangular PMMA phantom for acquiring the majority of the data. Although this was corrected using $B_{\text{PMMA}}^{\text{st}}$, human patients are comprised of soft tissue, bone, and air, and a more thorough investigation of the scattering properties of these materials would be helpful to estimate skin dose more accurately. Another limitation was the use of a single support pad for both systems; previous work has shown a relationship between TTF and support pad thickness.^20^ Vendors typically quote the equivalent aluminum thickness (mm Al Eq.) of the patient support, but this concept does not account for the effect of kVp, added Cu filtration, and the materials’ scatter properties.

## 5 CONCLUSION

Fluoroscopic skin dose may be calculated using the presented KCFs for under‐table x‐ray beam geometries, and the BSFs for lateral beam geometries. Kerma resulting from primary beam transmission, patient support forward scatter, and patient material backscatter underestimate the dose by up to 18% at the surface of 20‐cm thick PMMA phantoms. Exam‐specific radiation event data from the RDSR are available for many fluoroscopic systems, and the analytical equations herein may be used for more automated implementations of skin dose calculations. With the increase in awareness of elevated air kerma rates during complex FGI procedures, this work may aid the clinical physicist in performing peak skin dose calculations, or enable the opportunity to automate the calculation and improve accuracy.

## CONFLICTS OF INTEREST

The authors have no relevant conflict of interest to disclose.

## References

[1] National Council on Radiation Protection and Measurements . Radiation protection for procedures performed outside the radiology department. 2000; NCRP Report No. 133.

[2] Wagner LK , Eifel PJ , Geise RA . Potential biological effects following high x‐ray dose interventional procedures. J Vasc Interv Radiol. 1994;5:71–84. 8136601 10.1016/s1051-0443(94)71456-1

[3] Balter S , Hopewell JW , Miller DL , Wagner LK , Zelefsky MJ . Fluoroscopically guided interventional procedures: a review of radiation effects on patients’ skin and hair. Radiol. 2010;254:326–341. 10.1148/radiol.2542082312 20093507

[4] Miller DL , Balter S , Cole PE , et al. Radiation doses in interventional radiology procedures: the RAD‐IR study: part II: skin dose. J Vasc Interv Radiol. 2003;14:977–990. 12902555 10.1097/01.rvi.0000084601.43811.cb

[5] National Electrical Manufacturer’s Association . X‐ray Equipment for interventional procedures with user quality control mode. NEMA XR. 2018;27(R2018).

[6] International Electrotechnical Commission . Medical electrical equipment –Part 2–43: particular requirements for the safety of x‐ray equipment for interventional procedures. 2000; IEC report 60601.

[7] Rana VK , Rudin S , Bednarek DR . A tracking system to calculate patient skin dose in real‐time during neurointerventional procedures using a biplane x‐ray imaging system. Med Phys. 2016;43:5131–5144. 27587043 10.1118/1.4960368 PMC4991993

[8] Khodadadegan Y , Zhang M , Pavlicek W , et al. Automating monitoring of localized skin dose with fluoroscopic and interventional procedures. J Digit Imaging. 2011;24:626–639. 20706859 10.1007/s10278-010-9320-7 PMC3138926

[9] Johnson PB , Borrego D , Balter S , Johnson K , Siragusa D , Bolch WE . Skin dose mapping for fluoroscopically guided interventions. Med Phys. 2011;38:5490–5499. 21992367 10.1118/1.3633935 PMC3195372

[10] National Council on Radiation Protection and Measurements . Radiation dose management for fluoroscopically guided interventional medical procedures. NCRP Report No. 168, 2010.

[11] Jones AK , Pasciak AS . Calculating the peak skin dose resulting from fluoroscopically guided interventions. Part I: methods. J Appl Clin Med Phys. 2011;12:231–244. 10.1120/jacmp.v12i4.3670 PMC5718743 22089023

[12] International Council on Radiation Units and Measurements . Tissue substitutes in radiation dosimetry and measurement. 1989; ICRU Report No. 44.

[13] International Council on Radiation Units and Measurements . Physical aspects of radiation. 1962; ICRU Report No. 10.

[14] Grosswendt B . Backscatter factors for x‐rays generated at voltages between 10 and 100 kV. Phys Med Biol. 1984;29:579–591. 6739543 10.1088/0031-9155/29/5/010

[15] Petoussi‐Henss N , Zankl M , Drexler G , Panzer W , Regulla D . Calculation of backscatter factors for diagnostic radiology using Monte Carlo methods. Phys Med Biol. 1998;43:2237–2250. 9725601 10.1088/0031-9155/43/8/017

[16] Wunderle K , Godley A , Liu Shen Z , Rakowski J , Dong F . Percent depth doses and x‐ray beam characterizations of a fluoroscopic system incorporating copper filtration. Med Phys. 2017;44:1275–1286. 28094856 10.1002/mp.12109

[17] Balter S , Schueler BA , Miller DL , et al. Radiation doses in interventional radiology procedures: the RAD‐IR study: part III: dosimetric performance of the interventional fluoroscopy units. J Vasc Interv Radiol. 2004;15:919–926. 15361559 10.1097/01.RVI.0000130864.68139.08

[18] Sandborg M . Comparison between lucite and water as a phantom material in medical radiology. Prog Nucl Energy. 1990;24:355–364.

[19] Geiser W , Huda W , Gkanatsios N . Effect of patient support pads on image quality and dose in fluoroscopy. Med Phys. 1997;24:377–382. 9089590 10.1118/1.597906

[20] DeLorenzo MC , Yang K , Li X , Liu B . Comprehensive evaluation of broad‐beam transmission of patient supports from three fluoroscopy‐guided interventional systems. Med Phys. 2018;45:1425–1432. 29431862 10.1002/mp.12803

[21] Computerized Imaging Reference Systems . Dosimetry verification phantoms data sheet (701 706 DS 080715). http://www.cirsinc.com/products/all/33/atom‐dosimetry‐verification‐phantoms/ accessed 2019‐02‐09.

[22] Barthel KU . Volume Viewer 2003. https://imagej.net/plugins/volume‐viewer.html Accessed August 1, 2019.

